# Gastrointestinal Stromal Tumors: Molecular Mechanisms and Targeted Therapies

**DOI:** 10.4061/2011/708596

**Published:** 2011-04-14

**Authors:** Erinn Downs-Kelly, Brian P. Rubin

**Affiliations:** Departments of Anatomic and Molecular Pathology, Cleveland Clinic, 9500 Euclid Avenue, L25, Cleveland, OH 44195, USA

## Abstract

Gastrointestinal stromal tumors (GISTs) are the most common mesenchymal neoplasms of the gastrointestinal tract and are diverse not only in their clinical behavior but also in their histologic appearance. GISTs are insensitive to conventional sarcoma chemotherapy and radiation. However GISTs are sensitive to small-molecule tyrosine kinase inhibitors as 85–90% of GISTs have *KIT* or *platelet-derived growth factor receptor alpha (PDGFRA)* mutations, which drive tumorigenesis. This review will briefly touch on the clinicopathological features of GIST, while the majority of the review will focus on the clinical and treatment ramifications of *KIT* and *PDGFRA* mutations found in GIST.

## 1. Background

The last twenty years have seen great advances in the understanding of gastrointestinal stromal tumors (GISTs), from identifying their typical immunohistochemical phenotype and the molecular alterations that drive these tumors to the knowledge of their biologic potential and the use of effective tryosine kinase inhibitor targeted therapy. GISTs are the most common mesenchymal neoplasms of the gastrointestinal tract, and although insensitive to conventional sarcoma chemotherapy and radiation, they have shown dramatic clinical response to targeted kinase therapy. Activating mutations in *KIT* or *platelet-derived growth factor receptor alpha (PDGFRA) *have been identified in up to 80% and 10% of GISTs, respectively, and these mutually exclusive gain-of-function mutations play a fundamental role in GIST development by constitutively activating tyrosine kinase receptors [1–6]. Imatininib mesylate (ST1571; Gleevec, Novartis, East Hanover, NJ) is a selective tryosine kinase inhibitor that targets KIT and PDGFRA. The original indication was for the treatment of metastatic or unresectable GISTs with patients showing clinical responses in up to 80% of cases [7]; current FDA-approved labeling includes use in the adjuvant setting following complete gross resection of GISTs [8].

GISTs may occur anywhere in the gastrointestinal tract but are most common in the stomach and small bowel (roughly 60% and 30%, resp.), while 10% arise in other parts of the gastrointestinal tract (esophagus, colon, and rectum), and a small percentage are extragastrointestinal, arising in the mesentery, omentum, retroperitoneum, or pelvis [9, 10]. Once thought to represent smooth muscle neoplasms [11–13], GISTs are now known to share features with interstitial cells of Cajal (ICC), based on ultrastructure findings and immunophenotyping [14–19]. ICC are present within the interstitium of the muscularis propria throughout the gastrointestinal tract and serve a pacemaker function by generating and propagating electrical slow waves of depolarization, effectively coordinating peristalsis [16–18, 20, 21]. The current hypothesis is that GISTs arise from either the ICC or from a common progenitor stem cell [22]. 

GISTs are heterogenous, both from a clinical and morphologic stand point. Clinically, GISTs range from a small incidental finding that is entirely benign to a larger symptomatic neoplasm that may behave aggressively with metastatic potential. Regardless of their clinical diversity, GISTs share common genetic alterations. As mentioned above, mutually exclusive mutations in *KIT* or *PDGFRA *have been identified in up to 80% and 10% of GISTs, respectively [1–5]. These mutations have been detected in small, incidentally identified GISTs, suggesting that they occur as an early event in tumorigenesis [23, 24]. The majority of these mutations are somatic; however, germline mutations have been identified in rare families [25–30]. Approximately 5%–10% of GIST patients will lack mutations in either gene although KIT kinase activation is identified even in the absence of the mutation [2]. More recently, a primary V600E *BRAF *mutation was found within 7% of adult GIST patients that lacked either *KIT *or *PDGFRA *mutations [31]. Morphologically, GISTs may be either spindle cell, epithelioid, or mixed spindle and epithelioid cell types [32]. Epithelioid and mixed cell type GISTs are most commonly encountered in the stomach compared to other gastrointestinal sites [33, 34]. CD117 (KIT), the product of the *KIT *gene, has been identified as a sensitive immunohistochemical marker of GISTs from all sites and is expressed in up to 95% of GISTs with expression seen in wild-type tumors as well [35]. Approximately 5% of GISTs do not express KIT [36, 37], and this subset of KIT-negative GISTs frequently contain a *PDGFRA* mutation [3]. Roughly 70% of GISTs will express CD34 [38, 39], 20%–30% are positive for smooth muscle actin, 5% may express some positivity for S100 protein, and 1%-2% are positive for desmin or keratin [14, 32, 35]. 

## 2. *KIT* and *PDGFRA*

*KIT *and *PDGFRA *reside on chromosome 4q12 [40] with both genes encoding homologous transmembrane glycoproteins [41, 42] that are members of a type III tyrosine kinase receptor family. These transmembrane proteins contain an extracellular/ligand binding domain (EC) with five immunoglobulin-like loops that function in ligand binding and dimerization. This EC domain is connected to a cytoplasmic domain by a transmembrane domain. The cytoplasmic domain is composed of a juxtamembrane (JM) domain and tyrosine kinase domain (TK1 and TK2) which contains an adenosine triphosphate binding site and a phosphotransferase region separated by a kinase insert [43]. The JM domain regulates KIT tyrosine kinase activity by inhibiting activity in the absence of KIT ligand [44]. 

In the normal state, KIT and PDGFRA bind their respective ligands (stem cell factor and platelet-derived growth factors), leading to the phosphorylation of signal transduction proteins that modulate cell proliferation and chemotaxis and inhibit apoptosis [45, 46]. The signal transduction pathways involved include the mitogen-activated protein kinase (MAPK), phosphatidylinositol 3′kinase (PI3K), and Janus kinase/signal transducers and activators of transcription (JAK/STAT) pathways [47]. These intercellular signaling pathways play an important role in the development and maintenance of various cells including the interstitial cells of Cajal, mast cells, melanocytes, and hematopoietic stem cells [15, 48, 49]. 

## 3. Mutations as Drivers of Tumorigenesis in GISTs

Primary mutations in *KIT* or *PDGFRA* are a driving force for tumorigenesis and are identified prior to exposure to a tyrosine kinase inhibitor, while secondary mutations develop during targeted treatment with a tyrosine kinase inhibitor and account for acquired inhibitor resistance. *KIT *or *PDGFRA *mutations may affect either the regulatory domain (EC or JM domains) or the enzymatic domain (TK1 and TK2) of the tyrosine kinase receptor [50]. Regardless of either a primary or secondary mutation, *KIT* and *PDGFRA* mutations seen within GISTs activate receptor tyrosine kinases, leading to constitutive phosphorylation and the subsequent continued activation of the downstream intercellular signaling cascade. 

The oncogenic role of mutational activation of KIT or PDGFRA kinases has been supported by familial GIST syndromes and animal studies. Familial GIST syndromes may arise from germline mutations in exon 8, exon 11, exon 13, and exon 17 of *KIT *and in exon 12 of *PDGFRA *[27, 28, 51–57]. All of these syndromes have a high penetrance with nearly every effected family member developing GISTs that are typically multiple [26, 28, 51, 52, 55, 57, 58], while other clinical findings appear to be dependent on the domain mutated. For example, mastocytosis, urticaria pigmentosa, and diffuse hyperplasia of ICC with progression to distinct GISTs have been associated with mutations involving the JM domain of KIT [25, 27], while mutations that affect the kinase domain essentially lack mastocytosis and urticaria pigmentosa [28–30]. Transgenic mouse models have been developed with “knock-in” *KIT *mutations at either exon 11 or exon 13 wherein the mice develop ICC hyperplasia and GISTs [59, 60]. These are similar to the mutations identified in human sporadic and familial GISTs and supports that KIT activation is central to the development of GISTs. 

## 4. Genotype-Phenotype Correlations

**Figure 1:**
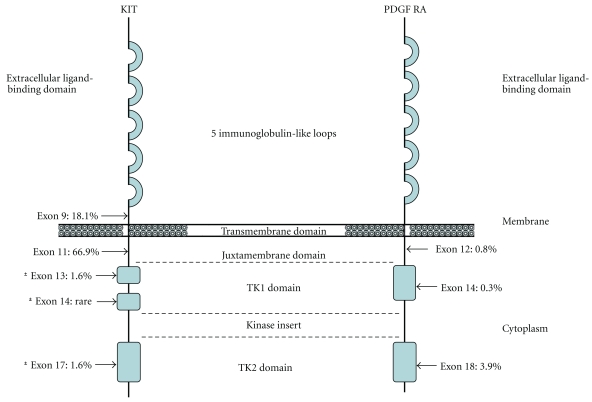
KIT and PDGFRA with locations and frequency of activating mutations in GISTs. Exons denoted with an *represent those most frequently involved by secondary mutations. Modified with permission from Heinrich et al. [3]. Copyright 2003 by the American Society of Clinical Oncology. All rights reserved.

Some important genotype-phenotype correlations have been identified in GISTs, not only pertaining to clinical behavior but also to the expected morphology and anatomic site of involvement for a given mutation. As mentioned previously, up to 80% of sporadic GISTs have mutations involving *KIT *[1–3, 6, 23] with the majority (approximately 75%) involving exon 11 of the *KIT *JM domain [44, 61–63] (Figure 1). The mutations cluster at either the 3′ or 5′ end of the exon. Mutations at the 5′ end most frequently include internal deletions [2, 64–68] and single nucleotide substitutions [2, 64–68], while duplications most commonly involve the 3′ end [69]. Although less common, internal tandem duplication mutations have been identified at the 3′ end of exon 11; clinically, these patients typically have gastric GISTs that follow an indolent course [68]. In comparison, an aggressive clinical course with a higher risk of recurrence and shorter survival has been noted in patients whose GISTs harbor deletions involving exon 11 [70–72]. This deletion has been shown to be an independent adverse prognostic factor [71] and when compared with GISTs that have other exon 11 mutations or are wild type, a poor disease-free survival has been associated with exon 11 deletions that specifically involve codon 557 and 558 [73–75]. The second most common *KIT *mutation site has been identified within exon 9 (distal extracellular domain); in this site, duplications are most commonly found [6]. Exon 9 mutations have been identified in roughly 10%–15% of sporadic GISTs and patients whose GISTs harbor this mutation commonly have small bowel involvement and a more clinically aggressive neoplasm [68, 75]. Mutations in exons 13 and 17 affect the tyrosine kinase domain and are seen in less than 5% of sporadic GISTS [6, 76]. Mutations involving these sites typically yield a spindle cell morphology and more frequent involvement of the small bowel than stomach [77].

Approximately 7% of GISTs harbor a mutation in *PDGFRA *[4, 5] with the majority being missense mutations identified in exon 18 affecting the TK2 domain [78, 79]. GISTs containing this mutation most commonly involve the stomach and have an epithelioid morphology [34, 78, 80]. *PDGFRA* exon 14 mutations are typically missense mutations that have been associated with epithelioid morphology, location within the stomach, and a favorable clinical course [78]. Rarely, mutations have been identified in the *PDGFRA* JM domain (exon 12), consisting predominately of point mutations, deletions, or deletion insertions [4, 78, 79]*. *In general, *PDGFRA * mutations are found within GISTs of the stomach and omentum, typically with epithelioid or mixed epithelioid/spindle cell morphology [34, 80–84]. 

## 5. Treatment and Emergence of Secondary Mutations

In regards to treatment, the main goal for a localized GIST is complete surgical resection with negative margins and preservation of an intact pseudocapsule. The tyrosine kinase inhibitor imatinib mesylate initially played a pivotal role in the management of metastatic or unresectable disease [7, 85–87] and is now used in the adjuvant setting following complete gross resection [8]. Imatinib is a small molecule tyrosine kinase inhibitor whose structure mimics ATP and binds competitively to the intracellular portion of KIT, inhibiting signaling. This molecule also targets PDGFRA. The clinical response to imatinib has been shown to be correlative with the particular *KIT *or *PDGFRA* mutation present. GISTs with exon 11 *KIT *mutations have shown the best imatinib response rates (up to 80% of patients with advanced disease either had partial response or achieved stable disease), while tumours with no *KIT *mutation or those with a *PDGFRA *D842V mutation were less likely to have a favorable or a sustained response to imatinib [3, 88]. Other studies have suggested that patients with exon 9 *KIT * mutations may benefit from the use of higher dose imatinib [89, 90]. 

Most GIST patients will develop resistance to imatinib after initially achieving a clinical response. This resistance is typically via secondary mutations that involve the kinase domain of *KIT * with these additional mutations found on the same allele as the primary mutation [91–94]. Some of these secondary kinase domain mutations are imatinib-resistant [91]. In these resistant tumours, the alternative kinase inhibitor sunitinib malate (SU11248 or Sutent, Pfizer, New York, NY) is being used [95–97]. This inhibitor targets several receptors including KIT, PDGFR, vascular endothelial growth factor receptors, and FLT3 and has shown clinical response in a variety of *KIT *and *PDGFRA *mutations; however, the development of sunitinib resistance is also an issue. Given this resistance, novel therapeutic strategies which target different aspects of intracellular signaling are being investigated. One strategy is to diminish KIT expression by inhibition of heat-shock protein (HSP)-90, a chaperone protein that aids in protein folding to stabilize KIT from degradation. The inhibition of HSP90 prevents the stabilization of KIT and leads to its degradation [98, 99]. 

## 6. Conclusions

GISTs are clinically and histologically heterogenous neoplasms that are driven by oncogenic *KIT *or *PDGFRA *mutations. Although the majority of GISTs show an initial clinical response to imatinib, the development of resistance to this tyrosine kinase inhibitor as well as to the alternative kinase inhibitor sunitinib is problematic. Future strategies to overcome resistance will likely have to target other intracellular signaling pathways.
